# Not One, but Many Critical States: A Dynamical Systems Perspective

**DOI:** 10.3389/fncir.2021.614268

**Published:** 2021-03-02

**Authors:** Thilo Gross

**Affiliations:** ^1^Helmholtz Institute for Functional Marine Biodiversity (HIFMB), Oldenburg, Germany; ^2^Institute for Chemistry and Biology of the Marine Environment (ICBM), Carl-von-Ossietzky Universität Oldenburg, Oldenburg, Germany; ^3^Helmholtz Center for Marine and Polar Research, Alfred-Wegener-Institute, Bremerhaven, Germany

**Keywords:** neural criticality, bifurcation, multi-criticality, critical brain, phase transition, criticality

## Abstract

The past decade has seen growing support for the critical brain hypothesis, i.e., the possibility that the brain could operate at or very near a critical state between two different dynamical regimes. Such critical states are well-studied in different disciplines, therefore there is potential for a continued transfer of knowledge. Here, I revisit foundations of bifurcation theory, the mathematical theory of transitions. While the mathematics is well-known it's transfer to neural dynamics leads to new insights and hypothesis.

## 1. Introduction

The critical brain hypothesis states that the brain operates in a state that is situated at or very near to a transition between qualitatively different dynamical regimes. Such “critical” states are thought to convey advantageous computational properties such as optimal information retention, signal detection and processing performance (Chialvo, 2010; Hesse and Gross, 2014; Zimmern, 2020).

The criticality hypothesis was first formulated based on the computational desirability of critical states (Chialvo, 2010) and a mathematical analogy between neural and earthquake dynamics (Herz and Hopfield, 1994). Subsequent works gradually build support for the hypothesis. For example Bornholdt and Rohlf (2000) showed that self-organized criticality can emerge from simple local rules, which was later confirmed in a more realistic models (Meisel and Gross, 2009; Kossio et al., 2018; Das and Levina, 2019). Beggs and Plenz (2004) provided early experimental evidence by demonstrating that *in-vitro* cultures of neurons sustain critical cascades of activity. Linkenkaer-Hansen et al. (2001), Kitzbichler et al. (2009), and Meisel et al. (2012) found signatures of criticality in MRI, MEG, and EEG recordings. More recently, Timme et al. (2016) confirmed the prediction that criticality maximizes information theoretic complexity and del Papa et al. (2017) shows that also learning behavior in recurrent neural networks leads to a state of criticality.

It has also been argued that brain could operate slightly below criticality. This is based on the analysis of experimental result on spike cascades (Priesemann et al., 2014) and is consistent with mathematical constraints on adaptive self-organization (Gross and Blasius, 2007; Kuehn, 2012; Droste et al., 2013). It has been argued that such an operation near critical states could allow the brain to control the desired degree of criticality (Wilting and Priesemann, 2014, 2018, 2019). Furthermore, in networks critical-like dynamics may be expected also in the neighborhood of the critical state in a so-called Griffith phase (Moretti and Munoz, 2013).

In the discussion of theoretical aspects of criticality many current authors resort to the toolkit of physics and its terminology and models, such as branching processes, correlation functions, critical exponents and the Ising model (Yaghoubi et al., 2018; Fontenele et al., 2019). However, critical phenomena can also be studied from the perspective of dynamical systems theory, which offers a complementary perspective to physical theory. Dynamical systems theory is the mathematical theory of transitions between dynamical regimes. Phase transitions then appear as so-called *bifurcations* of system-level dynamical variables. In neuroscience bifurcation-based methods are widely used in the study of smaller-scale neural networks but are not often deeply discussed in the context of neural criticality (although, see Meisel and Gross, 2009; Kuehn, 2012; Droste et al., 2013).

In this paper I present a mathematical view of neural criticality, The mathematics is relatively elementary and much of the material presented here can be found in introductory textbooks to bifurcation theory, e.g., Kuznetsov, 2004. However, several insights that can be gained by leveraging this angle are, to my knowledge, presently not utilized in the study of brain criticality. Thus it is worthwhile to bridge the gap between the neural and mathematical literature. Below I have tried to provide a simple and accessible introduction to the most relevant parts of bifurcation theory.

One particular benefit of mathematics is that it deals gracefully with unknowns. As this ability extends to working with unknown models, the use of mathematics allows the researcher to make statements about criticality that hold irrespective of the specific model under consideration.

Some highlight are as follows: In section 2.1, we revisit the origin of power law behavior and critical slowing down that gives critical states the ability to retain memory of perturbations. Thereafter in section 2.2, we illustrate why critical states can be super sensitive to parameter change. In section 2.3, we take a closer look at super-sensitivity and find that remaining close to a super-sensitive state places strong constraints on the dynamics. This is further explored in the subsequent section, starting with section 2.4, where we discuss the transcritical bifurcation (the criticality of the SIS model). While we find that it may play some role in neural systems, it provides less benefits than other bifurcations. This lends weight to the hypothesis that the criticality observed *in-vitro* may be of a different form than the criticality observed *in-vivo* (Kanders et al., 2017). In section 2.5, I show that pitchfork bifurcations (the criticality of the Ising model), is an unlikely scenario for neural criticality as it requires a specific symmetry. By contrast, in section 2.6, we discover that the Hopf bifurcation (the criticality of the Kuramoto model) has several advantageous properties that make it a particularly attractive scenario for neural criticality. In this type of bifurcation, information would be encoded by the presence or absence of oscillations in populations of neurons, which agrees well with empirical evidence. Finally in section 2.7, I discuss that high-dimensional parameter spaces have on criticality. This points to some radical perspectives: Critical states of the brain are likely not isolated points but part of a large high-dimensional subset of parameter space, which could allow the brain to explore different parameter regions while remaining critical. It could also lead to multi-critical states, corresponding to bifurcations of high codimension, where the brain is critical in many different ways.

## 2. Results

To gain insights it is useful to study a series of simple but general models. By keeping the models simple we make sure that the results we seek are easy to compute and intuitive to understand. By keeping them general we make sure that they are widely applicable and do not hinge on specific assumptions.

Consider a generic dynamical system of the form

(1) $$\begin{array}{l} \dot{x}=f\left(x,p\right), \end{array}$$

where *x* is a dynamical variable and *p* is a parameter. For example we can imagine *x* to be the overall level of activity in the brain and *p* to be the average excitability. The dot on the left hand side denotes a time derivative. So, the change of excitation in time is described by some function *f* of the current excitation *x* and the parameter *p*. In the following we will explore what properties of *f* would be advantageous for information processing.

Let us assume that over some time (and in absence of external stimuli) the excitation will approach a steady homeostatic level, which we will call x^*^. By definition a system that is in the *steady state* remains there indefinitely unless parameters are changed or it is subject to an external perturbation. That means in the state x^*^ there is no further change of *x*, which we can express as

(2) $$\begin{array}{l} f\left(x^{*},p\right)=0. \end{array}$$

Although the model is very minimal, we can use it to study how dynamical systems respond to perturbations. Consider what happens after some external force pushes the variable *x* out of the steady state x^*^, such that

(3) $$\begin{array}{l} x=x^{*}+\delta , \end{array}$$

where δ is the deviation from the steady state caused by the perturbation. We assume that this deviation is initially small, but grows or diminishes in time according to the dynamics of the system. Substituting Equation (3) into Equation (1) we can write

(4) $$\begin{array}{l} \frac{\text{d}}{\text{d}t}\left(x^{*}+\delta \right)=f\left(x^{*}+\delta ,p\right), \end{array}$$

where we have indicated the time derivative as d/d*t* instead of using the dot. Because the steady state x^*^ is constant in time, its time derivative vanishes, allowing us to return to the simpler notation,

(5) $$\begin{array}{l} \overset{∙}{\delta }=f\left(x^{*}+\delta ,p\right). \end{array}$$

To make further progress we need one mathematical tool: The Taylor expansion (James, 2015). The idea of a Taylor expansion is that we can approximate the function *f* by

(6) $$\begin{array}{l} f\left(x^{*}+\delta ,p\right)=f\left(x^{*},p\right)+\delta f_{\text{x}}\left(x^{*},p\right)+\frac{1}{2}\delta^{2}f_{\text{xx}}\left(x^{*},p\right)+\ldots \end{array}$$

where we used roman indices to indicate derivatives. So *f*_x_ is the derivative of *f* with respect to *x* and *f*_xx_ is the second derivative of *f* with respect to *x*.

While the Taylor expansion formula has an infinite number of terms on the right hand side, these terms include higher and higher powers of δ. If δ is a small number, say 0.01 then δ^2^ = 0.0001 is even smaller, and δ^3^ = 0.000001 is smaller yet. Hence, the terms in the Taylor formula represent smaller and smaller corrections.

If δ is sufficiently small then we can get an arbitrarily precise approximation by ignoring all but the first non-zero Taylor term. The first term *f*(x^*^, *p*) is always zero by virtue of Equation (2), hence in general the second term $\delta f_{rmx}\left(x^{*},p\right)$ is the one we need to keep. We are left with

(7) $$\begin{array}{l} f\left(x^{*}+\delta ,p\right)=\underset{=0}{\underset{︸}{f\left(x^{*},p\right)}}+\delta f_{\text{x}}\left(x^{*},p\right)+\underset{\approx 0}{\underset{︸}{\frac{1}{2}\delta^{2}f_{\text{xx}}\left(x^{*},p\right)+\ldots }}. \end{array}$$

Substituting the remaining term into Equation (5) we find

(8) $$\begin{array}{l} \overset{∙}{\delta }=\delta f_{\text{x}}\left(x^{*},p\right). \end{array}$$

This equation tells us that the speed at which the deviation changes is proportional to the size of the current deviation. If *f*_x_ is less than zero, the change counteracts the current deviation such that we return to the steady state. By contrast if *f*_x_ is greater zero then the deviation grows over time.

Equation (8) is a so-called separable differential equation and thus can be solved by the method of separation of variables (James, 2015). The result is the size of the perturbation as a function of time

(9) $$\begin{array}{l} \delta \left(t\right)=\delta \left(0\right)\exp f_{\text{x}}t. \end{array}$$

Here we have omitted the argument (x^*^, *p*) behind the *f*_x_ for simplicity. The solution shows that starting from the initial perturbation, δ(0), the deviation of system from the steady state grows or declines exponentially in time. Specifically, we observe an exponential growth if *f*_x_ > 0 and an exponential decline if *f*_x_ < 0. In the former case, the system is fundamentally unstable; any small perturbation launches it into dynamics that lead away from the steady state, so finding the system in this state at all seems implausible. In the latter case the state is stable, but the exponential return after a perturbation means that the memory of the perturbation is lost from the system exponentially fast.

The reasoning above illustrates a fundamental dilemma. The system cannot operate in an unstable state, because the very instability of the state precludes it from remaining there. By contrast the system can be in a stable state indefinitely, but the very stability of this state means that any information received is quickly lost from the system because the system returns to its previous state exponentially fast.

### 2.1. Origin of Power Laws and Critical Slowing Down

Let's explore what happens just at the boundary between stability and instability, i.e., in a *critical state*. In such a state we have *f*_x_ = 0. This means that the second term in the Taylor expansion (Equation 7) vanishes, so we can no longer argue that the third term is negligible by comparison. Instead we keep the third term,

(10) $$\begin{array}{l} f\left(x^{*}+\delta ,p\right)=\underset{=0}{\underset{︸}{f\left(x^{*},p\right)}}+\underset{=0}{\underset{︸}{\delta f_{\text{x}}\left(x^{*},p\right)}}+\frac{1}{2}\delta^{2}f_{\text{xx}}\left(x^{*},p\right)+\underset{\approx 0}{\underset{︸}{\ldots }}. \end{array}$$

Substituting the remaining term into Equation (1) gives us

(11) $$\begin{array}{l} \overset{∙}{\delta }=\frac{\delta^{2}f_{\text{xx}}}{2} \end{array}$$

Now the speed at which the deviation changes is proportional to the square of the current size of the deviation. Solving the equation with separation of variables yields

(12) $$\begin{array}{l} \delta \left(t\right)=\frac{2}{\frac{2}{\delta \left(0\right)}-f_{\text{xx}}t} \end{array}$$

**Figure 1:**
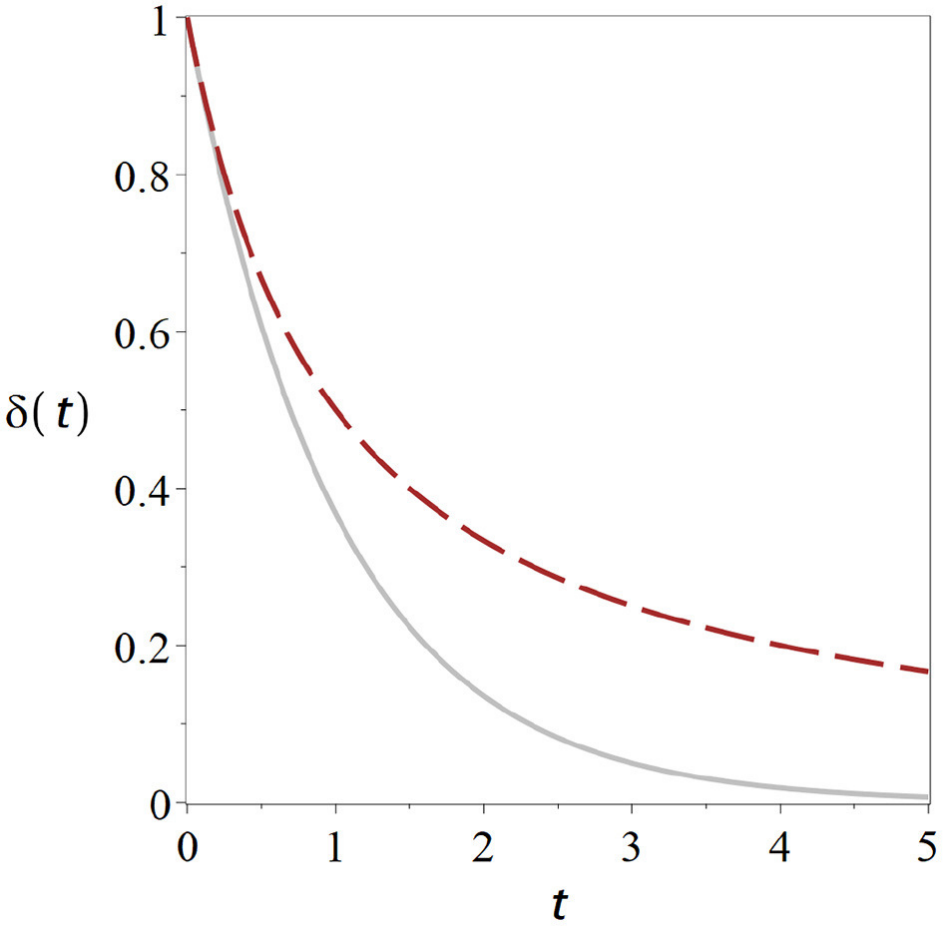
Critical states retain memory of perturbations. Curves show the return to the homeostatic state after a perturbation for a non-critical state (solid gray) and for a critical state (dashed black). The distance δ from the homeostatic level declines significantly slower for the critical state. Parameters have been chosen such that the curves start from the same initial perturbation with identical slope Exponential. Exponential return: exp (−*t*) (cf. Equation 9). Geometric Return: 2/(2 + 2*t*) (Equation 12).

The term *f*_xx_*t* in the denominator increases linearly in time, so after a sufficiently long time it will be much greater than δ(0). This means in the long run the δ(0) in the equation becomes negligible and the system behaves like 1/*t*. Instead of rapid exponential decline we now have a much slower geometric return to the stationary state (Figure 1). Hence, information about the perturbation is retained much longer in the system, and thus potentially long enough for slower, higher-order mechanisms of information retention to be set in motion.

The geometric return observed in the critical state is the cause of the widely-discussed phenomenon of *critical slowing down* (van Nes and Scheffer, 2007): Picture a system which is subject to small perturbations from time to time. We start our system in the stable regime, where it returns to the steady state exponentially fast after a perturbation. If we change the parameter we may observe that the exponential return gets slower and slower until eventually it becomes a geometric return at the point were stability is lost. So the recovery from perturbations slows down as we approach criticality.

Physically speaking δ(*t*) ~ 1/*t* is a power-law, although the power is 1 in this case. This power-law in the response to perturbations is the root cause of some of the power-laws that are often observed at criticality. For example if there is some noise present that causes repeated small perturbations the 1/*t* responses to each of the perturbations add up to a power-law in the systems power spectrum.

This is a nice result but there is still a problem: The system will only return the to the steady state after perturbations in a certain direction. All is well if δ(0) and *f*_xx_ have opposite signs. However if *f*_xx_ and δ(0) have the same sign then there will be a time when the denominator in Equation (12) is zero and as we approach this point the deviation becomes arbitrarily large. Of course we won't expect infinite excitation to occur in the real world; after all, our model is only valid for small deviations from the steady state. Nevertheless the result shows that certain perturbation lead to a dramatic departure from the steady state, so the state is unstable.

Below we describe two ways out of this stability-sensitivity dilemma in sections 2.4, 2.6, respectively.

### 2.2. Sensitivity to Parameters

So far we have presented inputs into the system as short perturbations of the system, an ecologist would call this a *pulse perturbation*. There is however also another way in which information may enter a system, the *press perturbation*, a sustained change of the environment that we can model as a change in parameters.

For example think of the parameter *p* as a sustained input into the system and ask how sensitive our steady state x^*^ is to this input. We can measure this in terms of the derivative

(13) $$\begin{array}{l} \frac{\text{d}}{\text{d}p}x^{*} \end{array}$$

The straight-d derivative that appears here denotes a differentiation where indirect effects are taken into account. By contrast the round-d partial derivative denotes a derivative where indirect effects are ignored.

A well-known trick to find this derivative is to differentiate the defining equation of the steady state Equation (2),

(14) $$\begin{array}{l} 0=f\left(x^{*},p\right) \end{array}$$

(15) $$\begin{array}{l} \frac{\text{d}}{\text{d}p}0=\frac{\text{d}}{\text{d}p}f\left(x^{*},p\right) \end{array}$$

(16) $$\begin{array}{l} 0=\frac{\partial }{\partial p}f\left(x^{*},p\right)+\left(\frac{\partial }{\partial x^{*}}f\left(x^{*},p\right)\right)\left(\frac{\text{d}}{\text{d}p}x^{*}\right) \end{array}$$

The differentiation of *f* in the second step results in two terms: The first captures the direct effect of change of *p* on *f*, whereas the second captures the indirect effect induced by the resulting change in x^*^. This second term is the product of the actual change in x^*^ and the response of *f* to a change in x^*^. Hence the derivative of x^*^ that we are looking for appears in the equation. Solving for it we obtain

(17) $$\begin{array}{l} \frac{\text{d}x^{*}}{\text{d}p}=-\frac{f_{\text{p}}}{f_{\text{x}}} \end{array}$$

where we have again used roman indices to denote the partial (round-d) derivatives.

Now consider what happens to Equation (17) if we consider the critical state from the previous section. Above we found that this state is characterized by *f*_x_ = 0, so that we have a infinitely sharp response to parameter change unless also *f*_p_ = 0. In the following we call this phenomenon super-sensitivity of the critical state.

Super-sensitivity is another attractive property of critical states: While systems normally responds proportionally to parameter change, a critical system can, at least potentially, show an abrupt out-of-proportion response. To understand when such a response is observed we have to examine the actual transitions more closely which we do in the next section.

### 2.3. A Closer Look at Super-Sensitivity

Critical states lie on the edges been qualitatively different types of behavior (*phases*) of a system. In the language of dynamical systems the transition between phases that takes place at the critical state typically corresponds to a *bifurcation*, a qualitative transition in the dynamics of the system. To get a better understanding of the transition we need to explore what happens in the bifurcations in more detail. Instead of just considering a perturbation of the state of the system *x*, we now consider also a small perturbation ρ of the parameter, such that

(18) $$\begin{array}{l} p=p^{*}+\rho \end{array}$$

where p^*^ is the *bifurcation point*, i.e., the critical parameter value where the bifurcation occurs.

To make progress we start again with our general system and Taylor expand with respect to both *x* and *p*:

(19) $$\begin{array}{l} \dot{x}=f\left(x,p\right) \end{array}$$

(20) $$\begin{array}{l} \;=f\left(x^{*},p^{*}\right)+f_{\text{x}}\left(x^{*},p^{*}\right)x+f_{\text{p}}\left(x^{*},p^{*}\right)p+\ldots \end{array}$$

(21) $$\begin{array}{l} \;=f_{\text{x}}\delta +f_{\text{p}}\rho \end{array}$$

In the second step we have used *f*(x^*^, p^*^) = 0 and omitted the arguments (x^*^, p^*^) for clarity.

The equation so far assumes that the two leading terms *f*_p_, *f*_x_ are non-zero such that we can neglect further terms (…) which contain higher powers of δ and ρ by comparison. While this is true in general, we are particularly interested in critical states where *f*_x_ = 0. This means the first term vanishes and we have to add some higher terms of the Taylor expansion instead

(22) $$\begin{array}{l} \dot{x}=f_{\text{p}}\rho +f_{\text{xx}}\delta^{2}/2 \end{array}$$

where the 2 appears due to the mechanics of the Taylor procedure. This expansion of the dynamical system is valid if

1. x^*^ is a steady state: *f*(x^*^, p^*^) = 0
2. The steady state is critical at p^*^: $f_{\text{x}}\left(x^{*},p^{*}\right)=0$
3. We can neglect higher order terms if δ an ρ are small: *f*_p_ ≠ 0, *f*_xx_ ≠ 0

The three conditions are of a very different nature. To satisfy the first two, the *stationarity condition* and the *bifurcation condition*, we must chose *x* and *p* exactly right to be in a steady state and to be at a bifurcation. The third condition is a *genericity conditions*, it will typically be met except in special cases.

To understand what happens in the bifurcation we can now solve the expanded equation for the steady state, i.e.,

(23) $$\begin{array}{l} 0=\dot{x} \end{array}$$

(24) $$\begin{array}{l} 0=f_{\text{p}}\rho +f_{\text{xx}}\delta^{2}/2 \end{array}$$

(25) $$\begin{array}{l} \delta =\pm \sqrt{-\frac{f_{\text{p}}\rho }{f_{\text{xx}}}} \end{array}$$

The result δ, shows us how much the steady state changes when we move the parameter *p* out of the critical point by an amount ρ. Equivalently we can write

(26) $$\begin{array}{l} x^{*}\left(p\right)=x^{*}\left(p^{*}\right)\pm \sqrt{-\frac{f_{\text{p}}}{f_{\text{xx}}}\left(p-p^{*}\right)} \end{array}$$

**Figure 2:**
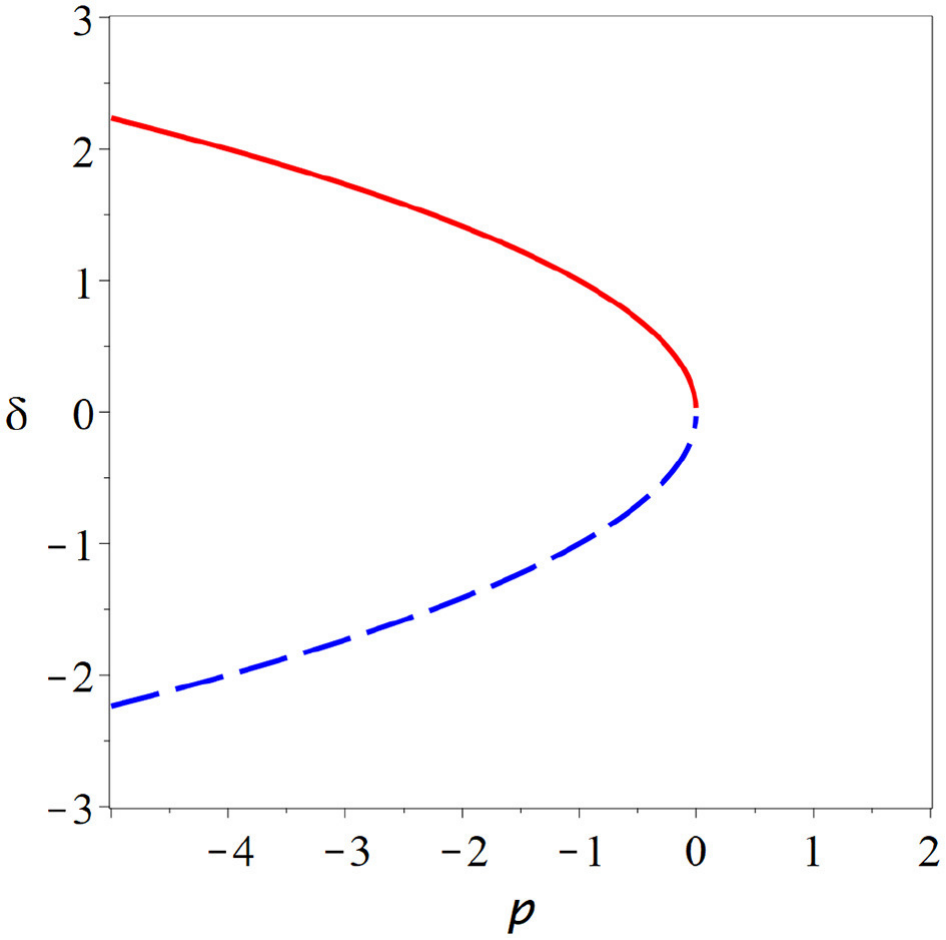
Fold bifurcation. As parameter *p* is changed two steady states (red solid, blue dashed) collide and annihilate (cf. Equation 26). At the bifurcation point, where the steady states meet the system is supersensitive to parameter change. But due to the instability of the bifurcation point and the lack of states beyond the bifurcation point, it seems implausible that the brain could remain in such a state.

The exact shape of the *branches* x^*^(*p*) depends on the values of the derivatives under the square root, but unless we are in a special case we always observe qualitatively the same picture. In the critical state two branches of steady states collide and annihilate each other (Figure 2).

At the point of collision the branches become vertical, which explains the super-sensitivity. One can show that for systems with one variable, one of the colliding steady states is stable whereas the other is unstable. Beyond the bifurcation both of the steady states involved have vanished, which means typically that the system departs the vicinity of the former steady states.

The bifurcation from Figure 2 is known under many names including fold bifurcation, saddle-node bifurcation, and turning point, among others. It depicts the generic behavior that we expect to see whenever we encounter a critical state in a system with one variable. However, it seems implausible that the brain would operate at such a bifurcation as the critical point is an unstable state and a small parameter variation is sufficient to destroy steady states entirely.

One could imagine that the brain has some mechanisms to stabilize it's operating point to a saddle-node bifurcation. However, if such mechanisms exist they are part of the same system, and by their presence may change the type of bifurcation or remove it entirely. Let us therefore instead look at some critical states in other types of bifurcations.

### 2.4. Epidemic-Like Criticality

The criticality hypothesis has long been attacked for requiring that one parameter is tuned exactly right such that the system is at a bifurcation point. This has become a much smaller concern as several models have shown that the brain could reliably self-tune its parameters to this operating point, using widely described mechanisms of synaptic plasticity (e.g., Bornholdt and Rohlf, 2000; Meisel and Gross, 2009). However, we now make an additional demand. Not only are the parameters tuned exactly to the bifurcation point, but also the system is such that we do not see a generic bifurcation, but a special case. However, there are some well-known scenarios where fundamental physical constraints and/or symmetries make sure that a system must always be in such a special case.

For example in many physical systems some variables cannot be negative by design. A prominent example is the prevalence of an epidemic, e.g., described by the SIS model (Anderson and May, 1979; Keeling et al., 2016). In an epidemic there is typically a steady state when the number of infected reaches zero, and this steady state cannot be perturbed in the negative direction as such a perturbation would be *unphysical*, leading to a negative number of infected.

Because the steady state at zero is there for a fundamental reason (if there are no infected nobody can become infected), the location of this steady state does not depend on parameters, and if it undergoes a bifurcation it cannot simply vanish as we would normally expect. Mathematically, the physical constraints on the steady state implies *f*_p_ = 0 and thus, by-virtue of the physics of the epidemic system, it's bifurcations at zero must always be of a special case.

For this case the Taylor expansion now reads

(27) $$\begin{array}{l} \dot{x}=f_{\text{px}}\rho \delta +f_{\text{xx}}\delta^{2}/2 \end{array}$$

Note that every term that contains more than one ρ and one δ is negligible in comparison to *f*_px_ρδ, moreover terms that contain more that two δ (e.g., δ^3^) are negligible compared to $f_{\text{xx}}\delta^{2}/2$ and all terms that contain no δ are zero due to the physics of the system.

This expansion is valid if

1. x^*^ is a steady state: *f*(x^*^, p^*^) = 0
2. The steady state is critical at p^*^: $f_{\text{x}}\left(x^{*},p^{*}\right)=0$
3. A genericity condition of the saddle-node bifurcation is violated *f*_p_ = 0 (also *f*_pp_ = 0, … )
4. We can neglect higher order terms if δ an ρ are small: *f*_px_ ≠ 0, *f*_xx_ ≠ 0

The third condition plays the role of an additional genericity condition for this type of bifurcation.

**Figure 3:**
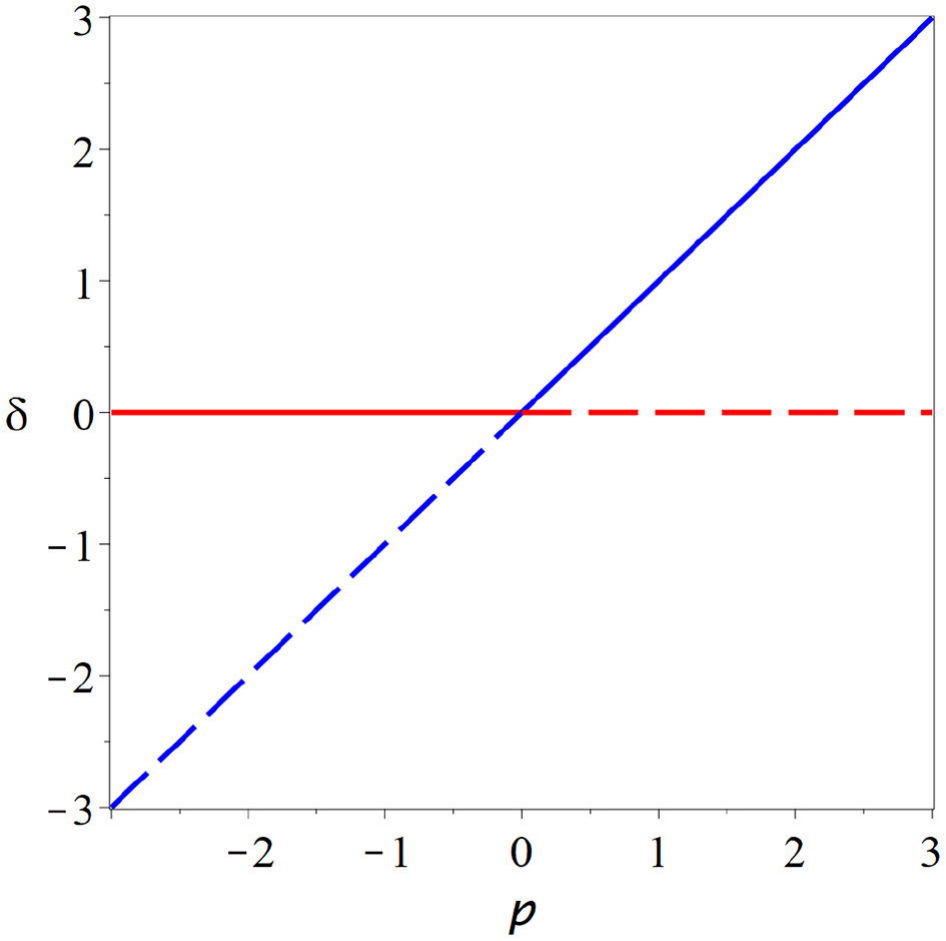
Transcritical bifurcation. In the transcritical bifurcation two branches of steady states (red, blue) intersect and exchange their stability. This type of bifurcation might play a role in the *in-vitro* neural networks, but several caveats make it appear as an unlikely operating point for the brain.

We can solve for the steady state

(28) $$\begin{array}{l} 0=f_{\text{px}}\rho \delta +f_{\text{xx}}\delta^{2}/2, \end{array}$$

which gives us two solutions, δ = 0 and

(29) $$\begin{array}{l} \delta =-\frac{f_{\text{px}}}{f_{\text{xx}}}\rho , \end{array}$$

a second branch that crosses the branch at zero in the bifurcation point. Stability analysis reveals that the branches exchange their stability in the bifurcation point (Figure 3). This *transcritcal* bifurcation is a typical scenario for the onset of epidemics. If the parameter is low enough, the disease-free state is stable, but once a threshold is crossed the disease-free state loses stability as a new steady enters the physical space in which the disease persists indefinitely.

**Figure 4:**
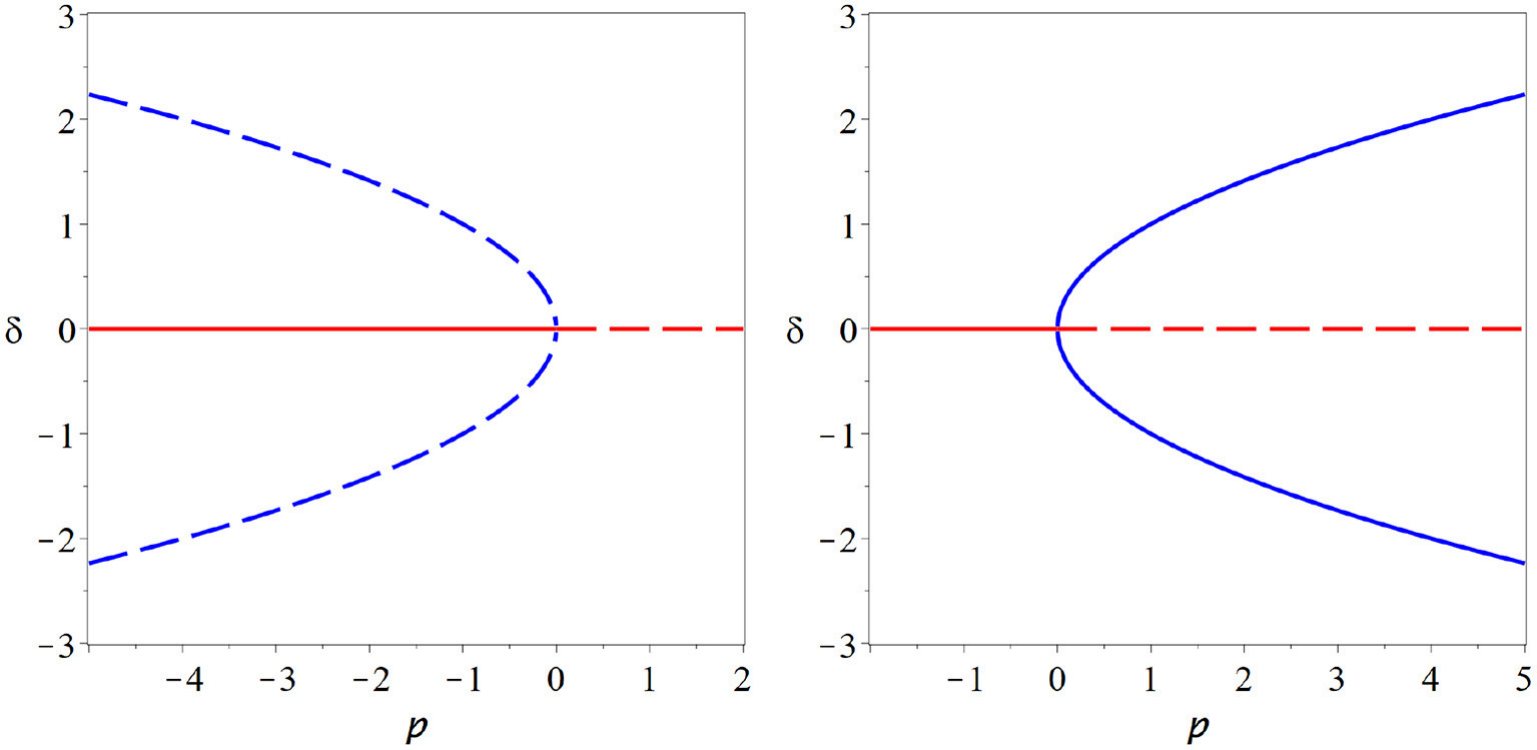
Pitchfork bifurcation. In this bifurcation a steady state (red) loses stability as either two additional branches of steady states (blue dashed) are destroyed (subcritical case, **left**) or two branches of stable steady states (blue solid) emerge (supercritical, **right**). A system could plausibly operate at a supercritical pitchfork and profit from persistent memory and super-sensitivity that this bifurcation conveys. However, the pitchfork bifurcation requires a special symmetry that is hard to motivate for the brain, it therefore is an unlikely candidate for the operating point of neural criticality.

Because the overall activity appears here as the order parameter of the bifurcation this is also the dimension in which computational benefits are reaped. It is therefore reasonable to expect this bifurcation to play a role when information is coded in terms of activitiy.

The transcritical bifurcation has some attractive features as a model for neural criticality. If we are willing to neglect spontaneous activity we can argue that the system should have a steady state at zero. Furthermore if the variable *x* represents a rate of spikes, we can argue that this variable can not be negative. Under these assumptions the state at a transcritical bifurcation is stable if *f*_xx_ < 0, and thus the system could plausibly remain there while profiting from the long memory that comes with criticality.

Note that the nature of the bifurcation has implications for information processing. If we are willing to accept that the brain operates at a transcritical bifurcation, then this would suggest that information is coded directly in terms of activity: After a perturbation causes increased activity, the system remains in a state of increased activity while slowly decaying back to the resting state where activity is zero.

There is indeed some evidence that points to transcritical-type criticality in the brain. The state at the transcritical bifurcation is characterized by activity cascades with branching ratio 1, which is in agreements with observations from *in-vitro* cultures (Beggs and Plenz, 2004; Hesse and Gross, 2014) and also direct measurements in life animals (Klaus et al., 2011; Hahn et al., 2017).

However, there are also some caveat regarding the transcritical bifurcation. It is subject to structural instability on which we discus in some more detail below. Additionally this bifurcation does not create super-sensitivity; because *f*_p_ = 0 the solution branches never become vertical (Figure 3). Thus this bifurcation scenario misses one of the two key features that make criticality attractive for computation.

In summary the transcritical bifurcation probably plays some role in systems of neurons. Particularly it is likely that this is the bifurcation that is encountered when one observed the onset of activity in neural networks and perhaps also in mature systems grown *in-vitro*. Moreover the observation of activity cascades and power-laws at in experiments supports this hypothesis. However, both evidence for other forms of information coding, and the caveats regarding transcritical bifurcations, suggest that other bifurcation scenarios also play significant and perhaps greater role for information processing in the brain.

### 2.5. Ising-Like Criticality

A very popular model system for criticality is the Ising model. The bifurcation that occurs in this model is the *pitchfork bifurcation*, another degenerate form of the fold bifurcation. In this case the degenerate bifurcation appears because the model is motivated by a physical system that has a mirror symmetry. Due to this symmetry all terms of the Taylor expansion that are derivatives of even order with respect to *x* must be zero. This implies *f*_p_ = 0 and also *f*_xx_ = 0 so both genericity conditions of the fold bifurcation are violated.

In this case the expansion becomes

(30) $$\begin{array}{l} \dot{x}=f_{\text{px}}\rho \delta +f_{\text{xxx}}\delta^{3} \end{array}$$

which is valid if

1. x^*^ is a steady state: *f*(x^*^, p^*^) = 0
2. The steady state is critical at p^*^: $f_{\text{x}}\left(x^{*},p^{*}\right)=0$
3. First fold genericity condition is violated: *f*_p_ = 0 (also *f*_pp_ = 0, … )
4. Second fold genericity condition is violated: *f*_xx_ = 0
5. We can neglect higher order terms if δ an ρ are small: *f*_px_ ≠ 0, *f*_xxx_ ≠ 0

Solving for the steady state in the steady state in this case reveals three branches: the zero solution δ = 0 and a pair of branches

(31) $$\begin{array}{l} \delta =\pm \sqrt{-\frac{f_{\text{px}}}{f_{\text{xxx}}}\rho } \end{array}$$

If *f*_px_ and *f*_xxx_ have the same sign these two branches exist only for ρ < 0, otherwise they exist only for ρ > 0. Furthermore one can show that if *f*_px_ > 0 then the steady state at zero is stable for ρ < 0 (and vice versa).

In the *subcritical* form of the pitchfork bifurcation *f*_xxx_ < 0 the non-zero branches are unstable. In the bifurcation point they collide with the stable branch at zero and vanish as the zero becomes unstable. This leads to a *catastrophic* bifurcation after which no stable steady state is left. By contrast in the *supercritical* from of the pitchfork bifurcation *f*_xxx_ the steady state at zero becomes unstable as two stable non-zero branches emerge.

The supercritical pitchfork bifurcation is in principle an attractive model for neuroscience as the critical state is stable and has the desirable characteristics of long-term memory of perturbations and super sensitivity to parameter change.

The major problem with this sort of bifurcation is that it is hard to motivate why such dynamics should occur in the brain. The bifurcation requires a perfect mirror symmetry which is easy to motivate for the physical Ising model (spin up and spin down states are thought to be exactly symmetrical) but is hard to justify in a biological system.

**Figure 5:**
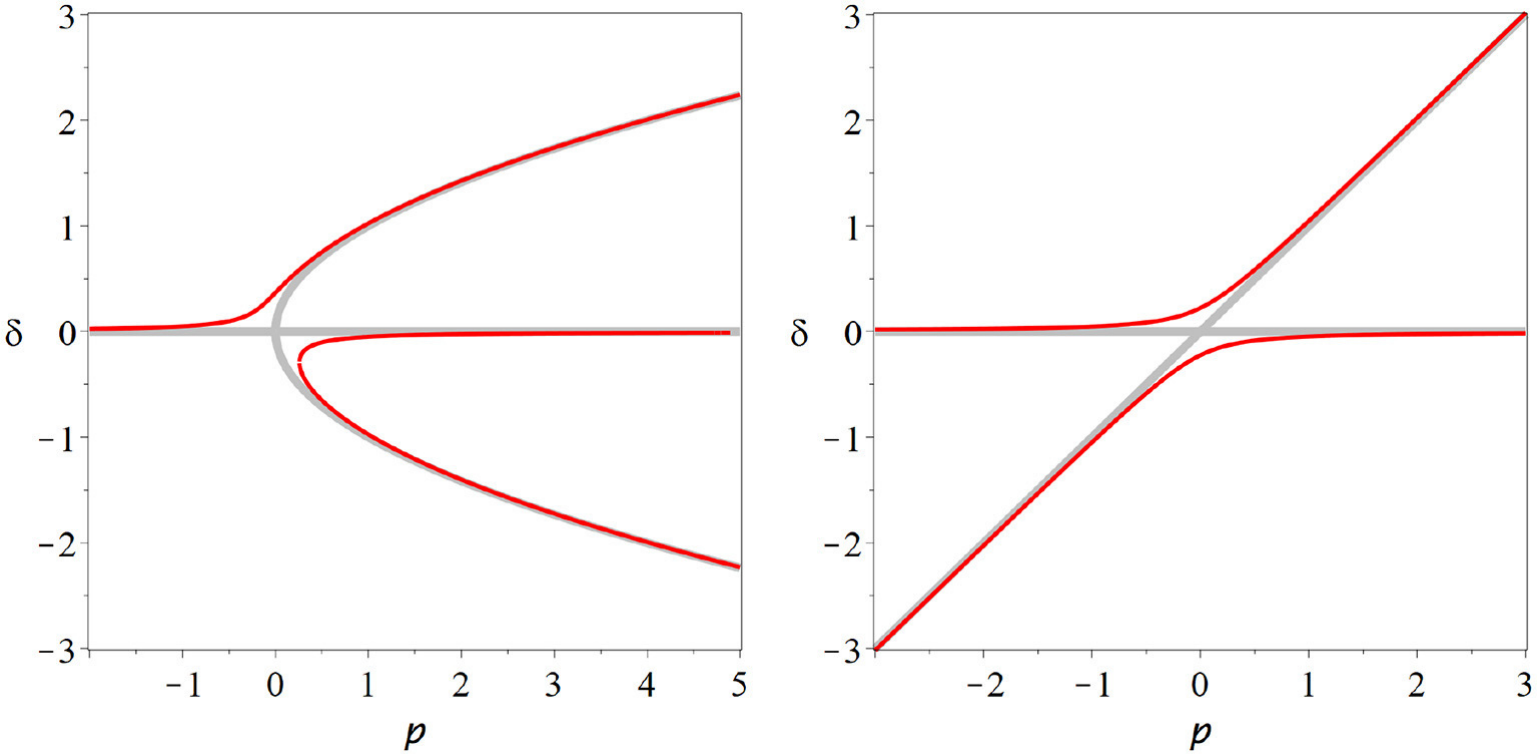
Structural instability. The pitchfork **(left)** and transcritical **(right)** bifurcations are degenerate bifurcations: To observed their characteristic bifurcation diagrams (thick gray lines) special cases particular symmetries must exist in a model. If we break these symmetries, e.g., by adding a low level of spontaneous activity, then the degenerate bifurcation revert back to the generic fold bifurcation, or no bifurcation at all (red lines).

All degenerate bifurcations, including transcritical and pitchfork suffer from structural instability (Figure 5). For example including even a low level of spontaneous activity destroys the transcritical bifurcation in SIS-type models model entirely. However, for multiple reasons we should not disregard degenerate bifurcations altogether. Also the transcritical bifurcation vanishes from the SIS model if spontaneous activity is included. However, it is replace by a region where the solution branch bends quickly, through not abruptly. This region of rapid change will retain some semblance to a critical state.

Moreover higher-level mechanisms may exist that drive the brain to degenerate bifurcations in a very similar way that to proposed primary self-organization to critical states (Seung, 1996; Feudel et al., 2018). For example Seung (1996) describes how neurons can approximate a degenerate line attractor, but also notes some caveats.

Even in absence of mechanisms that create degenerate situations over a broad range of operating conditions, the pitchfork bifurcation may play a role in information processing in decision making. Making decisions is only a challenge when different options appear almost exactly equally desirable. However, this equal desirability creates exactly the symmetry needed for pitchfork bifurcations.

For example the occurrence of a pitchfork bifurcation has been well-documented in collective decision making in fish faced with a binary choice task (Couzin et al., 2011).

The pitchfork-in-decision scenario is interesting because we get *criticality on demand*. The need for a decision, creates a situation in which the prerequisite symmetry for pitchfork criticality exists. The system can then be critical and profit from the super-sensitivity that this entails. Once the decision has been make the symmetry is broken, potentially leaving the system non-critical in this respect. This on-demand criticality is possible due to the difference between the slow timescale on which the need for the decision arises and the fast timescale of decision making processes.

### 2.6. Criticality at the Onset of Oscillations

In the previous sections, we have gone on a fairly exhaustive trawl of bifurcations of systems with one variable, but from the perspective of neuroscience none of the bifurcations scenarios we found was completely satisfactory. Of course there are other, even more degenerate bifurcations that we haven't discussed. For example there could be a transcritical-like bifurcation where three branches intersect or a pitchfork-like bifurcation where one branch splits into five. But essentially these are variations on a theme. If we want super sensitivity and memory in a stable critical state in a system we so far need to impose mirror symmetry.

An elegant way out of the dilemma is to consider systems with more than one variable. All the bifurcations that occur in systems with one variable (fold, transcritical, pitchfork,.) also occur in two-variable systems. Moreover, another type of long-term behavior is possible: sustained oscillations. The geometrical object in variable space on which such oscillations take place, a cycle, can undergo the same bifurcation as a steady state in one-variable systems, hence there can be a fold bifurcation of cycles, in which a stable and an unstable cycle collide and annihilate. However, all of these bifurcations present us with the same dilemma as the bifurcations in one-variable systems.

A genuinely new bifurcation of two-variable systems that does not have an equivalent in one-variable systems is the Hopf bifurcation. In this bifurcation a cycle emerges from (or is destroyed upon collision with) a steady state. The mathematical analysis of this bifurcation is slightly more complicated, hence I omit the expansion here (it can be found in Kuznetsov, 2004), but the key idea in this analysis is that one can transform the two variables of the system (say, *x, y*) near the bifurcation to obtain an angle and radius variable,

(32) $$\begin{array}{l} r=\sqrt{{\left(x-x^{*}\right)}^{2}+{\left(y-y^{*}\right)}^{2}} \end{array}$$

(33) $$\begin{array}{l} \phi =\arctan\left(\left(y-y^{*}\right)/\left(x-x^{*}\right)\right) \end{array}$$

so *r* denotes the distance from the original steady state and the ϕ denotes the angle between the state of the system and the steady state. In these new variables the dynamical equations close to the bifurcation are captured by an expansion of the form

(34) $$\begin{array}{l} \overset{∙}{\phi }=a \end{array}$$

(35) $$\begin{array}{l} \dot{r}=br\rho +cr^{3} \end{array}$$

where *a*, *b*, and *c* are constants that arise from derivatives of the dynamical equations (similarly to *f*_x_ etc.) and ρ is again our control parameter that measures the distance to the bifurcation point. Considering these equations we can see that the angle changes with a continuous angular velocity *a*. The equation for the radius is more interesting: The radius equation always has a stationary solution at *r* = 0. Even in this state the angle is constantly changing, but because the radius is 0 our original variables *x, y* remain stationary–this solution is our initial steady state. Stability analysis shows that it is stable if *bρ* ≤ 0 and unstable otherwise.

**Figure 6:**
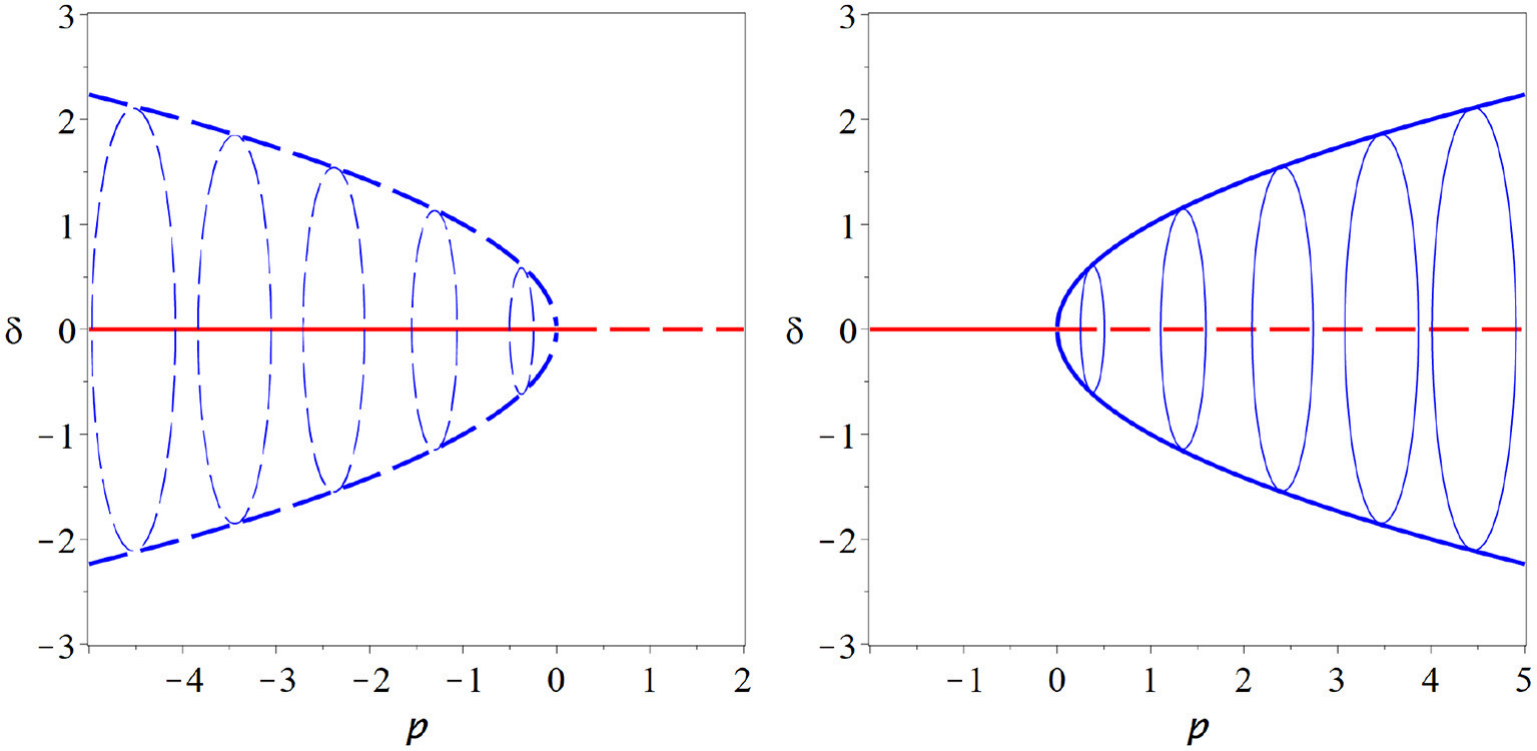
Hopf bifurcation. The Hopf bifurcation marks the onset of at least transient oscillation in a dynamical system. It is in many ways similar to a pitchfork bifurcation and shares many of its attractive features. In contrast to the pitchfork, a stable cycle is created in the Hopf bifurcation (supercritical case, **right**) or an unstable cycle vanishes (subcritical case, **left**). Moreover, the Hopf bifurcation is a generic bifurcations and thus does not require hard-to-justify assumptions. These properties make it very attractive as an potential operating point for the brain (thin blue lines indicate some examples of the cycle, coming out of the plane of the paper).

Looking closely at the equation for *r* we note that this equation has the same form as the expansion of the pitchfork bifurcation, so at ρ = 0, where the initial steady state loses its stability, two new stationary solutions of *r* emerge. One is at negative radius and hence unphysical, whereas the other is at a positive radius. Due to the constant progression of the angle ϕ this stationary point of *r* is a cycle in the *x, y* coordinates (Figure 6).

The Hopf bifurcation inherits many properties from the pitchfork bifurcation. Like the pitchfork it has a subcritical and a supercritical form. In the supercritical case a stable limit cycle emerges as the initial steady state loses stability. The existence of stable dynamics on both sides of the bifurcation allows a system to operate in the vicinity of the bifurcation. Moreover, like the pitchfork, the Hopf bifurcation offers long memory of perturbations (perturbations create long-lasting oscillations) and super sensitivity to parameter change (quick rise of oscillation amplitude when the bifurcation point is crossed).

In contrast to the pitchfork, the Hopf bifurcation is a generic bifurcation. Thus we don't have to introduce hard-to-justify assumptions to observe this bifurcation.

In a complex system Hopf bifurcations typically occur when the microscopic parts of the system synchronize. At this point preexisting oscillations on the micro-level transition from oscillating asynchronously to a synchronous mutually reinforcing state such that detectable system-level oscillations are produced. Such a scenario is very plausible for the brain as the individual neurons already have oscillatory characteristics and have been illustrated in models (Brunel, 2000).

In this case the variables *x* and *y* typically relate to the microscopic variables of the individual oscillators. For example in a model *x* could be the number of neurons that are just spiking whereas *y* is the number of neurons that are currently refractory.

Seen in this light the Hopf bifurcation becomes an order-disorder transition in which disordered phases of oscillators become organized. It has been pointed out that systems can self-organized robustly using simple local rules (Droste et al., 2013). Moreover, spike-timing dependent plasticity observed in neurons is a rule that has the right characteristic to drive the system to such a transition (Meisel and Gross, 2009).

The assumption that the brain (or parts of the brain) operate at a Hopf bifurcation is consistent with information coding in terms of synchrony. The experimental evidence for this type of information coding (e.g., de Charms and Merzenich, 1996) thus lends further weight to this hypothesis.

Among all conceivable bifurcation scenarios on stationary states, and cycles the Hopf bifurcation is the only scenario that offers a stable operating point, super-sensitivity and structural stability. These properties make it extremely attractive for the use in computational systems.

Operating at a Hopf bifurcation point provides the advantageous properties of long information retention and super-sensitivity to parameter change. At the same time very plausible mechanism exist by which the brain could self-organize to this bifurcation and remain there indefinitely. These advantageous features highlight models of synchronization, such as networks of Kuramoto oscillators as promising conceptual models for neural dynamics.

### 2.7. The Critical Hypersurface

So far we have only studied bifurcations in diagrams with one parameter axis. The same is true for almost all papers that discuss bifurcations in the context of neural criticality. However, let's break this convention and consider what happens in systems with two or more parameters.

The bifurcations that we discussed so far are so-called bifurcations of *codimension 1*. This means that the bifurcations have a single bifurcation condition. To find the bifurcation we must change the parameters until we find a parameter set where the bifurcation condition is met. If we change one parameter we might eventually meet the condition and observe the bifurcation at a specific parameter value.

Mathematically speaking we can say if we have a one-dimensional parameter space (i.e., a parameter axis) then codimension-1 bifurcations occur in a zero-dimensional subset (i.e., specific points in parameter space).

Now suppose we have two parameters *p*_1_, *p*_2_. In the two-dimensional parameter space the bifurcation condition becomes a function of both parameters. Because we only have only one bifurcation condition we can (in general) satisfy it already by setting one of the parameters, say *p*_1_ to the right value. That means (in a typical scenario) that for every value of *p*_2_ we can find the bifurcation at some value of *p*_1_: The bifurcation points fill a curve in the two-dimensional parameter plane.

The existence of this curves of critical point allows the system to move around in parameter space, while remaining at criticality all the time.

A system that has at least two parameters could self-organize to criticality and then start to drift on a curve of critical states. As we drift on the curve we can even encounter further bifurcations, so-called codimension-2 bifurcation points. In such a point the system is then critical in two different ways. For example it is conceivable that we reach a point where a Hopf and a transcritical bifurcation happen at the same time (a degenerate Takens-Bogdanov bifurcation). In a neural system that could be a point where we observe an onset of spontaneous activity (trancritical bif.) and at the same time the onset of synchronization of this activity (Hopf). Likewise we could imagine a higher-codimension bifurcation where the onset of oscillations takes place at the same time as changes in the number of synchronized clusters. Such higher a bifurcation would be very attractive for information processing.

In the real world much more than two parameters could be relevant. If our system has *d* parameters the bifurcation points of a codimension-1 bifurcation completely fill a (*d*−1)-dimensional subspace. We can say that the form *hyper-surfaces*. In a high-dimensional parameter space the existence of these hypersurfaces gives a self-organizing system potentially a huge parameter space to move around in while staying critical.

It is interesting to ask how many parameters exist in the brain. So far there are only partial answers to this question. On the one hand we might go down the list of network properties that are known to affect network dynamics: The include average connectivity, it's second moment, the spectral radius, the clustering coefficient and various other motif counts. While it is not clear that all of these affect the network dynamics independently we can say that there are at least several of these topological parameters are commonly found to affect dynamical processes on networks.

On the other hand, we could ask how many parameters are necessary to characterize the network structure of the brain completely. In this case the answer is at least one per synapse, which means the effective dimensionality *d* of the parameter space could be as high as the number of synapses.

So the best of the author's knowledge we can say that the effective dimensionality of the parameter space of the brain is somewhere between tens and billions of parameters. Any answer in this range means that the brain is not confined to a single critical point in parameter space, but has in-fact a huge high-dimensional space to explore, in which it could plausibly sit at the threshold of many different bifurcations at the same time. A particularly intriguing picture is to imagine the brain poised at the critical points of a large number of different Hopf bifurcations, each corresponding to the synchronization of a different community of neurons.

In summary the potentially very high effective dimensionality of the brain opens up some startling perspectives. We should not think of the brain as a system that sits stationarily in one point where a certain codimension-1 bifurcation happens. Instead the brain might be at some very high-codimension that is critical in many (and potentially very many) ways at the same time. Alternatively, mechanism of plasticity could take it on a self-organized journey that explores a high-dimensional critical hyper-surface. In the authors opinion, the most likely scenario is that both of these phenomena, drift on a critical manifold and high-codimension multi-criticality occur simultaneously in the brain.

## 3. Conclusions

In this paper I have reviewed some relatively basic and well-known dynamical systems theory, which nevertheless has profound implications for neural dynamics. Along the way we have discussed some side attractions (stability constraints, origins of power laws and critical slowing down, absence of super sensitivity in the transcritical bifurcation). However, perhaps the two most important messages are the ones that are hinted at in the title. There are many critical states in at least two ways:

1. There are many different types of bifurcations that can occur at critical points. And potentially all of the ones discussed here play some role in neural information processing. At the same time the supercritical Hopf bifurcation seems uniquely attractive for cortical information processing because it is the only scenario that allows criticality in a stable steady state, while providing super-sensitivity without requiring a specific degeneracy.
2. Even for a specific type of bifurcation, one should not think of the critical point as an isolated point in parameter space. In a high dimensional parameter space the critical points fill an almost equally high-dimensional hyper-surface. This means that mechanisms of self-organization can explore a large parameter space while maintaining criticality. It also means that the system can reach high-codimension points where the system is simultaneously critical in several, and potential many, different ways.

Particularly the second point highlights the need for future theoretical work to explore how self-organized critical systems drift on critical manifolds and assess the consequences of multi-criticality for information processing. So far such dynamics in high-dimensional parameter spaces remains largely unexplored.

## Data Availability Statement

The original contributions presented in the study are included in the article/supplementary material, further inquiries can be directed to the corresponding author/s.

## Author Contributions

The author confirms being the sole contributor of this work and has approved it for publication.

## Conflict of Interest

The author declares that the research was conducted in the absence of any commercial or financial relationships that could be construed as a potential conflict of interest.
